# Supplementary material for: Resolving the full spectrum of human genome variation using Linked-Reads
Source: Genome Res. 2019 Apr;29(4):635–45. doi: 10.1101/gr.234443.118 (PMC6442396; doi:10.1101/gr.234443.118)
Supplement: Supplemental Material [file supp_gr.234443.118_Supplemental_longranger-2.2.zip › longranger-2.2.tar.bz2!/lr2.2-public/lib/stan/cmdstan/stan/doc/api/html/agrad_2rev_2functions_2binary__log__loss_8hpp_source.html]

Stan: src/stan/agrad/rev/functions/binary\_log\_loss.hpp Source File


|  |  |
| --- | --- |
|  | Stan  2.5.0  probability, sampling & optimization |


- Main Page
- Related Pages
- Namespaces
- Classes
- Files

- File List
- File Members

All Classes Namespaces Files Functions Variables Typedefs Enumerations Enumerator Friends Macros Pages

- src
- stan
- agrad
- rev
- functions

binary\_log\_loss.hpp

Go to the documentation of this file.

1 #ifndef STAN\_\_AGRAD\_\_REV\_\_FUNCTIONS\_\_BINARY\_LOG\_LOSS\_HPP

2 #define STAN\_\_AGRAD\_\_REV\_\_FUNCTIONS\_\_BINARY\_LOG\_LOSS\_HPP

3

4 #include <valarray>

5 #include <stan/agrad/rev/var.hpp>

6 #include <stan/agrad/rev/internal/v\_vari.hpp>

7 #include <stan/math/constants.hpp>

8 #include <stan/math/functions/log1p.hpp>

9

10 namespace stan {

11  namespace agrad {

12

13  namespace {

14  class binary\_log\_loss\_1\_vari : public op\_v\_vari {

15  public:

16  binary\_log\_loss\_1\_vari(vari\* avi) :

17  op\_v\_vari(-std::log(avi->val\_),avi) {

18  }

19  void chain() {

20  avi\_->adj\_ -= adj\_ / avi\_->val\_;

21  }

22  };

23

24  class binary\_log\_loss\_0\_vari : public op\_v\_vari {

25  public:

26  binary\_log\_loss\_0\_vari(vari\* avi) :

27  op\_v\_vari(-stan::math::log1p(-avi->val\_),avi) {

28  }

29  void chain() {

30  avi\_->adj\_ += adj\_ / (1.0 - avi\_->val\_);

31  }

32  };

33  }

34

69  inline var binary\_log\_loss(const int y, const stan::agrad::var& y\_hat) {

70  if (y == 0)

71  return var(new binary\_log\_loss\_0\_vari(y\_hat.vi\_));

72  else

73  return var(new binary\_log\_loss\_1\_vari(y\_hat.vi\_));

74  }

75

76  }

77 }

78 #endif

stan::agrad::var::vi\_

vari \* vi\_

Pointer to the implementation of this variable.

**Definition:** var.hpp:40

v\_vari.hpp

var.hpp

log1p.hpp

stan::agrad::var

Independent (input) and dependent (output) variables for gradients.

**Definition:** var.hpp:27

stan::agrad::binary\_log\_loss

fvar< T > binary\_log\_loss(const int y, const fvar< T > &y\_hat)

**Definition:** binary\_log\_loss.hpp:15

constants.hpp

stan::agrad::log

fvar< T > log(const fvar< T > &x)

**Definition:** log.hpp:15

stan::agrad::log1p

fvar< T > log1p(const fvar< T > &x)

**Definition:** log1p.hpp:16


---

[ Stan Home Page ]

*© 2011–2014,
Stan Development Team.*
